# Microplastics in seawater: sampling strategies, laboratory methodologies, and identification techniques applied to port environment

**DOI:** 10.1007/s11356-020-07783-8

**Published:** 2020-02-06

**Authors:** Laura Cutroneo, Anna Reboa, Giovanni Besio, Franco Borgogno, Laura Canesi, Susanna Canuto, Manuela Dara, Francesco Enrile, Iskender Forioso, Giuseppe Greco, Véronique Lenoble, Arianna Malatesta, Stéphane Mounier, Mario Petrillo, Ruben Rovetta, Alessandro Stocchino, Javier Tesan, Greta Vagge, Marco Capello

**Affiliations:** 1 DISTAV, University of Genoa, 26 Corso Europa, I-16132 Genoa, Italy; 2 DICCA, University of Genoa, 1 Via Montallegro, I-16145 Genoa, Italy; 3 ERI – European Research Institute Onlus, 24/d Via Pinelli, Turin, Italy; 4 Laboratoire MIO, University of Toulon, CS 60584, 83041 Toulon CEDEX 9, France

**Keywords:** Microplastics, Seawater monitoring, Sampling devices, Visual sorting, Analytical identification, Port environment

## Abstract

The European Interreg Italy–France 2014–2020 Maritime Project SPlasH! (Stop to Plastics in H_2_O!) focused on the study of microplastics (MPs) in the marine port environment to evaluate their presence, abundance, and mechanisms of diffusion to the open sea. In the framework of this project, a worldwide review of 74 studies was carried out, providing an overview of MP investigation techniques, focusing on sampling strategies, laboratory methodologies, and identification of MPs collected in seawater, and specifically evaluating their applicability to the marine port environment. Nets were the most commonly used device for MP surface sampling, but their use can be difficult in narrow spaces within the port basins, and they must be coupled to discrete sampling devices to cover all port basins. In the laboratory, density separation (NaCl, ZnCl_2_, NaI, sodium lauryl sulfate (SLS)), filtration (polycarbonate, polyamide, glass, cellulose, ANOPORE inorganic membrane filters), sieving, visual sorting, and digestion methods (acidic, enzymatic, alkaline, oxidative) were used to separate MPs from seawater. Digestion becomes essential with water samples with great inorganic and organic loads as deriving from a port. Although many studies are based only on visual MP identification under a microscope, analytical identification techniques unequivocally determine the particle nature and the identity of the plastic polymers and are necessary to validate the visual sorting of MPs. Fourier-transform infrared spectroscopy (FTIR) is the most used analytical identification technique.

**Electronic supplementary material:**

The online version of this article (10.1007/s11356-020-07783-8) contains supplementary material, which is available to authorized users.

## Introduction

Plastic is a synthetic organic compound, derived from polymerization processes of organic and inorganic raw materials, such as carbon, silicon, hydrogen, oxygen, and chloride, which can be extracted from oil, coal, or natural gas (Ivar do Sul and Costa 2014). Plastic represents one of the main wastes in seas and oceans (de Lucia et al. 2014). This is due to the widespread use of plastic, which started around six decades ago (Thompson et al. 2004; Barnes et al. 2009), and it has grown continuously, with a global production of more than 348 million tons in 2017. Asia (50.1%) and China in particular (29.4%) are the largest producers of plastics, followed by Europe (18.5%) and countries that are part of the North American Free Trade Agreement (NAFTA, 17.7%) (PlasticsEurope 2018).

Plastic released into the marine environment can have land-based origins (e.g., household or personal care products that reach the sea mainly through waste treatment) or marine-based origins (e.g., nylon nets used for fishing) (de Lucia et al. 2014; Duis and Coors 2016). Due to different physical mechanisms of transport, in particular, currents and tides, plastics are widespread in all marine environments, from the poles to the equator (Derraik 2002; Ivar do Sul and Costa 2014; Lusher et al. 2015; Cincinelli et al. 2017).

The wide plastic diffusion in the marine environment becomes even more important considering plastics with small dimensions (from 1 μm to 5 mm are called microplastics (MPs); Gago et al. 2018). MPs tend to accumulate on the surface of the water column, but they are also transported vertically to the bottom through different mechanisms (Wang et al. 2016). Indeed, during their permanence in seawater, the density of the plastic particles can increase, for example, due to biofouling (Cózar et al. 2014; Wang et al. 2016). Moreover, turbulence generated by strong wind events can determine the mixing of the sea surface layer and, consequently, the redistribution of plastics within this layer (Collignon et al. 2012). In addition, MPs can concentrate different contaminants on their surface, such as persistent organic pollutants (POPs), and tend to be easily ingested by aquatic organisms, thus entering the food chain carrying contaminants with them. For this reason, they endanger marine organisms and possibly affect human health since MPs have been found in different foodstuffs, including seafood (Karbalaei et al. 2018; Rist et al. 2018).

Many efforts are spent in MP study and MPs are part of the national and international laws. For example, the Honolulu Strategy was developed by the United Nations Environment Programme (UNEP) and the National Oceanic and Atmospheric Administration (NOAA), and it provides approaches to minimize plastic waste and to reduce marine debris (Xanthos and Walker 2017). Several initiatives across the world have banned plastic bags and microbeads such as the Canadian Environmental Protection Act (Schnurr et al. 2018). At the European level, the Marine Strategy Framework Directive (MSFD/2008/56/EC) of the European Commission (EC) establishes a framework for community action in the field of marine environmental policy. It sets the achievement of the Good Environmental Status (GES) by 2020 as the main target, which shall be assessed based on 11 descriptors (European Commission 2008; Gago et al. 2016). MPs fall within descriptor number 10, concerning Marine Litter and are a parameter that needs to be monitored following two criteria: criterion “D10C2 - Primary: The composition, amount and spatial distribution of micro-litter on the coastline, in the surface layer of the water column, and in seabed sediment, are at levels that do not cause harm to the coastal and marine environment”; criterion “D10C3 - Secondary: The amount of litter and micro-litter ingested by marine animals is at a level that does not adversely affect the health of the species concerned” (European Commission 2017). In 2018, the first European strategy on plastics was adopted, establishing a ban of non-reusable and non-recyclable plastic packaging by 2030 (Schnurr et al. 2018).

Despite the great attention placed on the study of MPs, a standard methodology for the MP study in terms of abundance, distribution, and effects on organisms is lacking. Thus, it is difficult to make comparisons between different areas and studies. In this context, the European Commission set up a Technical Group on Marine Litter (TGML) under the MSFD Common Implementation Strategy. One of the documents developed by this organization is the Guidance on Monitoring of Marine Litter in European Seas, which provides the member states of the European Union with recommendations to follow the same strategies on MP investigation in a marine environment (Galgani et al. 2013). Other two research groups that dealt with finding common indications for the study and monitoring of MPs in the marine environment are the Joint Group of Experts on the Scientific Aspects of Marine Environmental Protection (GESAMP) and JPI-Oceans that published recently two guides for MP study in the ocean (Gago et al. 2018; GESAMP 2019).

The European Interreg Italy–France 2014–2020 Maritime Project SPlasH! (Stop to Plastics in H_2_O!; http://interreg-maritime.eu/web/splash) has entered the European panorama of MP study and focused on the MPs investigation inside the marine port environment. This study aimed to analyze common MP sampling strategies, laboratory methodologies, and identification techniques to identify different methods that have been specifically used for seawater analyses all over the world and find the appropriate devises and methodologies for applications inside ports.

**Fig. 1:**
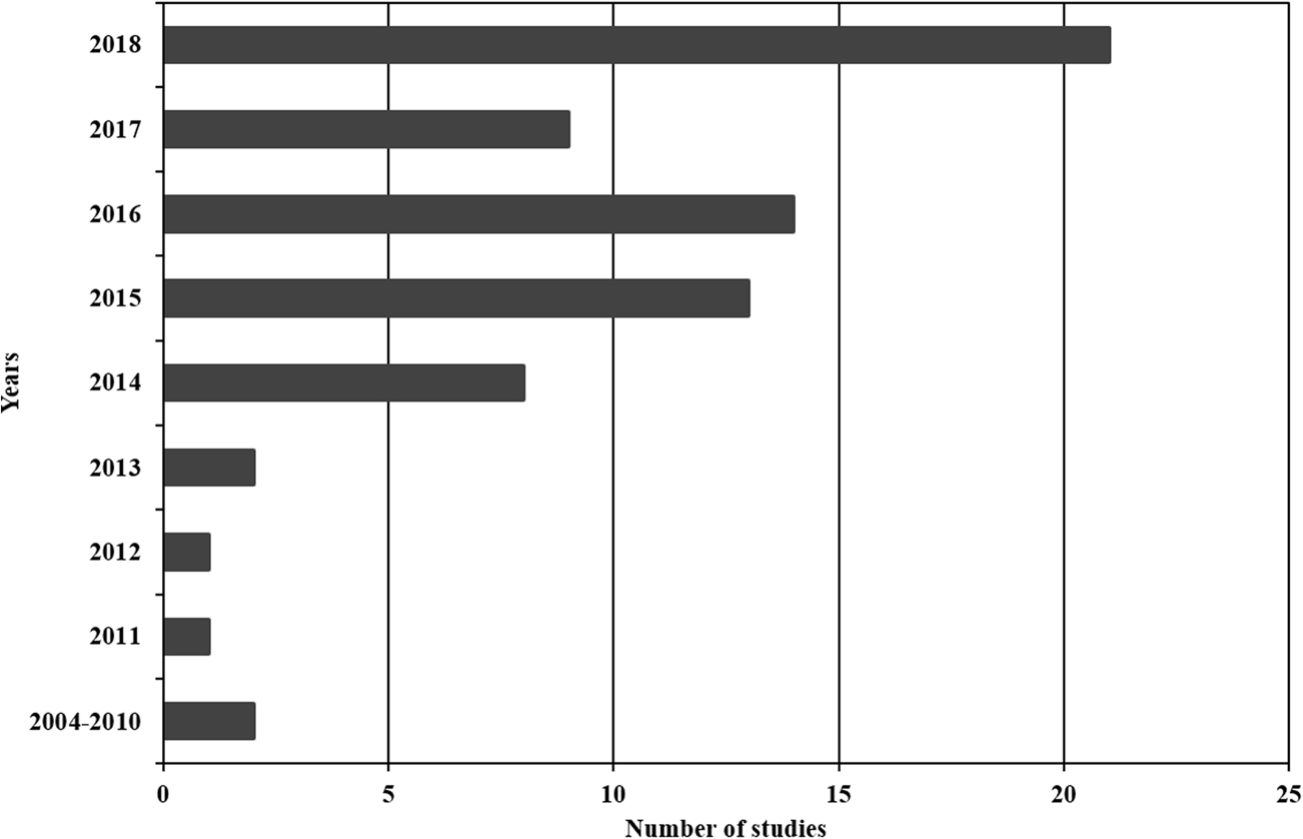
Evolution in the published studies on microplastics in seawater from 2004 to 2018

**Fig. 2:**
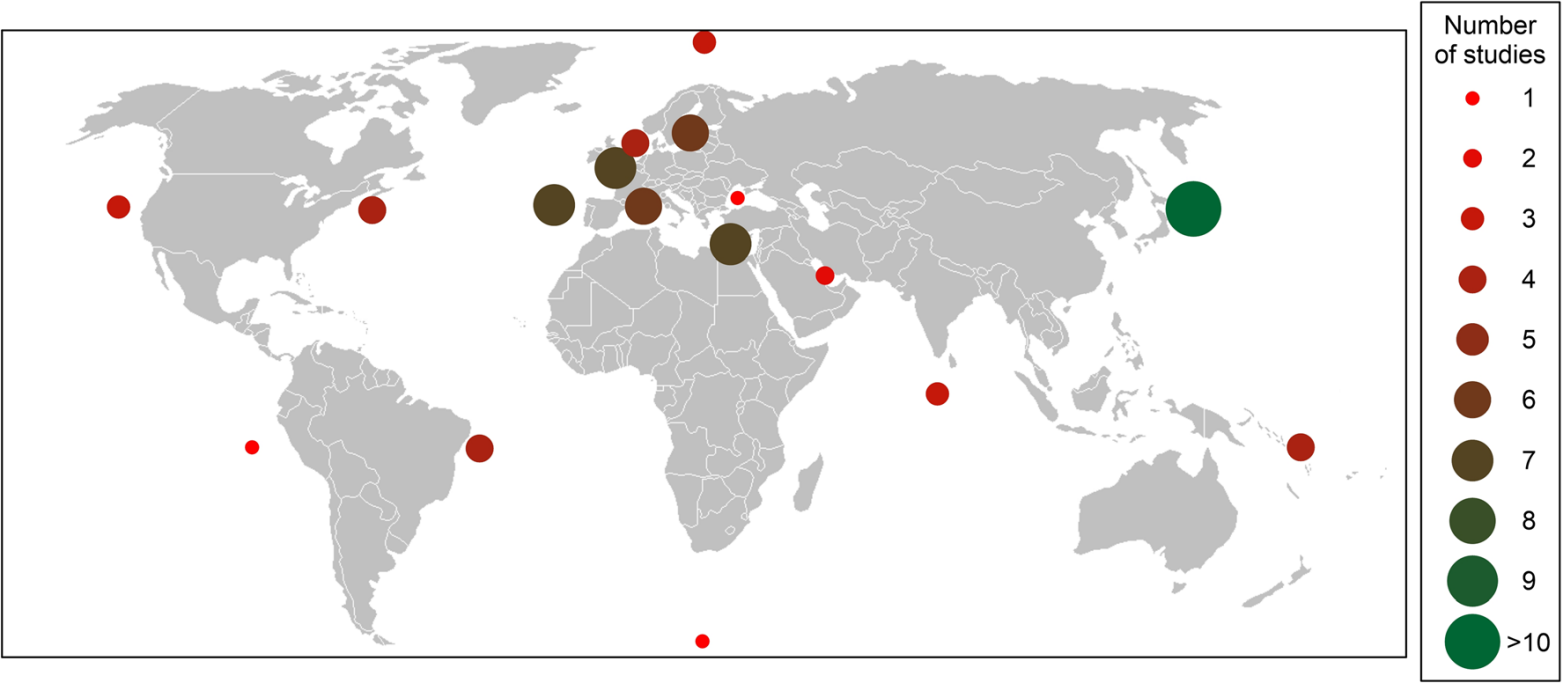
Distribution of areas where the studies analyzed in the present review are conducted and numbers of studies (from 1 to > 10) for each area showed by the colored circles

The following literature review was carried out from July 2018 to January 2019 using databases, such as Science Direct and Google Scholar and selecting only research studies on MPs in seawater. The term “microplastics” was used for the first time in 2004 (Thompson et al. 2004; Hidalgo-Ruz et al. 2012), which why the research articles found do not date back prior to this year. A total of 74 research articles were found (complete reference list in Online Resource 1): most publications are from 2018, with 81% of all literature published in the last 5 years (Fig. 1), and most studies were in the Northern Hemisphere instead of the Southern Hemisphere, as already noted by Gago et al. (2018) (Fig. 2).

## Microplastic sampling

MP sampling strategies can be distinguished in bulk and volume-reduced samplings (Hidalgo-Ruz et al. 2012). Bulk sampling consists of collecting the entire volume of seawater without reducing it during this process, while the extraction of MPs occurs in a laboratory. Volume-reduced sampling means that each sample is taken, reducing its volume during the sampling process and preserving only the portions of the samples that are useful for further processing in a laboratory. This strategy provides for the extraction of MPs from seawater samples filtered with nets during the sampling process.

MPs in seawater are sampled using different kinds of devices. Based on the device used, it is possible to collect different layers of the water column, from the surface layer (from 0 to 1 m depth; Song et al. 2015) to the bottom layer (Lima et al. 2014). Sampling devices can be divided into three categories: non-discrete sampling devices (nets and pumping systems), discrete sampling devices (Niskin bottles, rosette, integrating water sampler [IWS], bucket, bottle, and steel sampler), and sampling devices of the surface microlayer (sieves and rotating drum sampler).

### Non-discrete sampling devices

#### Nets

**Fig. 3:**
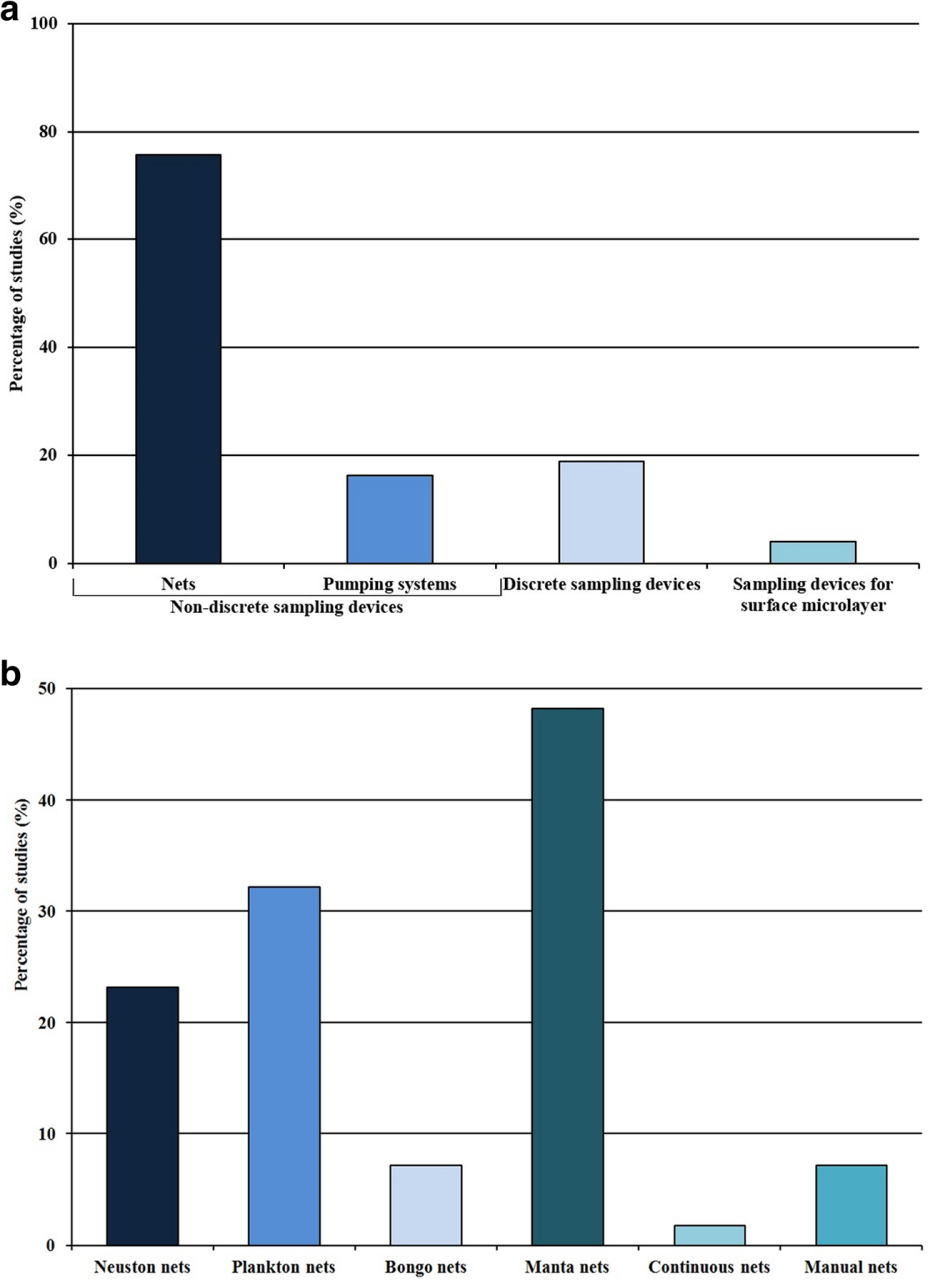
(a) Number of reviewed studies expressed in percentage (%) in which different sampling devices are used. (b) Number of the reviewed studies expressed in percentage (%) in which different types of net are used; maximum percentage shown in the *y*-axis is equal to 50 instead of 100 to be able to appreciate the difference between each histogram

Nets have been designed for collecting plankton, but they recently started being used for MP sampling. In general, nets allow for sampling large volumes of water, from the surface to the bottom layer (Lima et al. 2014; Kang et al. 2015). Nets are the most used devices for MP sampling and their application occurs in 56 out of 74 analyzed studies (76%) (Fig. 3a). In these 56 studies, different kinds of nets were used, including a neuston net, plankton net, manta net, continuous net, and manual net (Fig. 3b). General information about the characteristics of the nets used in the analyzed studies is shown in Table 1.

**Table 1:** Characteristics of devices used for MP sampling in the studies analyzed in the present study

Nets					
Net mesh size (μm)	Area (m^2^) of the rectangular/diameter (m) of the circular opening of net	Length of net (m)	Towing time (minutes)	Towing speed (knots)	References (complete reference list in Online Resource 1)
Neuston nets					
64–350	0.1–1.2 m^2^	1–4	3–30	1.5–4	[1], [5], [22], [25], [27], [28], [34], [35], [51], [52], [53], [59], [72]
Neuston DiSalvo nets					
300	0.3 m^2^				[22]
Neuston net designed by Syakti et al. (2018)					
5000; 1000; 500; 300; 100	0.4 m^2^			1	[63]
Plankton nets					
50–500	0.2–0.6 m	1.5–2.5	3–30	1–5	[7], [10], [11], [13], [25], [29], [31], [33], [39], [41], [47], [67]
Plankton WP2 nets					
90–200	0.2–0.6 m	2.6	3–20	1.5–4	[2], [4], [14], [27], [30], [32]
Bongo nets					
150–500	0.2–0.6 m	15	2–5	3–5	[6], [9], [31], [60]
Manta nets					
300–500	0.1–0.61 m^2^	2–4.5	5–240	1–5	[4], [8], [15], [17], [22], [23], [24], [26], [31], [32], [36], [43], [48], [49], [51], [55], [56], [61], [65], [68], [69], [70]
High-speed Manta net					
333	0.1 m^2^	4.5	60		[44]
Suitcase Manta net					
333	0.1 m^2^			1–3	[54]
AVANI trawl					
335	0.1 m^2^	4	60	5	[22]
Longhurst Hardy Plankton Recorder (LHPR)					
335			30	4	[27]
Manual nets					
20–80	0.2–0.3 m	0.6			[36], [38], [47], [56]
Others					
Device			Volume (L)		
Continuous Plankton Recorder (CPR) coupled with a 280 μm mesh screen					[66]
Niskin Bottle			10		[3]
			30		[3]
Bucket					[3], [16], [42]
Multi Water Sampler SlimLine 12					[3]
Jar/Bottle/Becker/Steel Sampler					[5], [19], [30], [31], [36], [50], [64], [73]
Integrated Water Sampler (IWS)					[64]
Pumping systems					[12], [18], [21], [37], [40], [43], [45], [46], [71]
Manta Ray					[20]
Rosette Sampler System					[16], [37]
Rotating Drum Sampler					[46]
PLastic EXplorer (PLEX)					[74]

Nets are deployed from the ship using structures (spinnaker, A-frame, etc.) that allow for displaying nets away from the ship to avoid bow wave and turbulence generated from the ship wake (Palatinus et al. 2015; Green et al. 2018). Nets are usually equipped with a flow meter for measuring the amount of water that has been filtered during the trawl (Norén 2007). If a flow meter is not present, the amount of filtered water can be calculated with the net opening size and the length of the transect, which in turn can be calculated as the distance between the starting and ending points (Eriksen et al. 2018). Nets may have a rectangular or circular opening (Gajšt et al. 2016; Figueiredo and Vianna 2018; Tunçer et al. 2018) with different dimensions (Table 1). The net aperture is composed of a rigid frame that maintains a continuous rectangular or circular net opening at the surface, while at the end there is a collecting jar where the sample is concentrated. Two wings, made of a mixture of resins, may be welded at the side of the aperture to keep the net floating on the surface or at a specific depth. Nets are usually built with plastic material (e.g., polyvinyl chloride (PVC)).

Nets are 1.5–4.5 m long (Gajšt et al. 2016; Maes et al. 2017) and they have variable mesh sizes (20–5000 μm; Khalik et al. 2018; Syakti et al. 2018) (Table 1). A 333-μm net mesh is most commonly used in the considered literature (Table 2). The use of these mesh sizes causes underestimation of the real number of MPs because the loss of the smaller sizes occurs. However, nets with smaller mesh sizes (e.g., 80 μm) are difficult to use because they can get clogged up, compromising the sampling process (Löder and Gerdts 2015), in particular in a port environment where suspended particles can be very concentrated. For this reason, it is also recommended to avoid collecting samples during phytoplankton blooms (Löder and Gerdts 2015). To overcome the underestimation of MP abundance, with a net, it is possible to combine a device (e.g., discrete sampling device) that allows for the specific evaluation of the small fraction of MPs (Sedlak et al. 2017). Otherwise, it is necessary to consider the underestimation due to the lack of information for smaller particles. Nets can be towed horizontally (Collignon et al. 2014), within the superficial layer or at greater depths, vertically from the bottom to the surface to sample the entire water column (Gorokhova 2015; Baini et al. 2018), or obliquely. In the case of horizontal sampling seawater, nets were towed along the horizontal transect within the surface layer for specific periods (3–240 min; Frias et al. 2014; Pan et al. 2019) at a known speed (1–5 knots; Eriksen et al. 2018; Green et al. 2018; Syakti et al. 2018). The speed of the vessels must be calibrated to avoid turbulence, which may influence the flow rate through the net opening and, consequently, the quality of the collected samples.

**Table 2:** Net mesh sizes used for sampling microplastics in the studies analyzed in the present review. Different net mesh sizes can be used during the same research. To simplify the table, references are here indicated with a number and the corresponding complete list is presented in the Online Resource 1

Net mesh size (μm)	Number of research studies	References [Online Resource 1]
20	1	[38]
50	3	[33], [36], [56]
64	1	[25]
80	2	[7,47]
90	1	[30]
100	1	[63]
120	1	[10]
135	1	[67]
150	2	[6,11]
180	1	[27]
200	9	[2], [4], [13], [14], [25], [31], [32], [53], [59]
280	1	[27]
300	8	[1], [22], [23], [28], [29], [31], [41], [63]
308	1	[48]
330	7	[4], [24], [36], [49], [55], [56], [65]
333	12	[8], [9], [15], [32], [43], [44], [51], [54], [61], [68], [69], [72]
335	5	[5], [22], [26], [27], [51]
350	3	[34], [35], [52]
355	1	[39]
400	1	[31]
450	1	[47]
500	5	[13], [17], [31], [60], [63]
1000	1	[63]
5000	1	[63]

After sampling, nets need to be rinsed from the outside, preferably with decontaminated water (e.g., Milli-Q water), to avoid sampling contamination. Hence, all sampled MP particles are gathered in the collecting jar. Then, the collecting jar is rinsed repeatedly using decontaminated water (e.g., Milli-Q water) and emptied into another jar. Each sample collected with a net is preserved in a jar until laboratory analysis. The net, once cleaned, can be used for the next sampling.

#### Pumping systems

Pumping systems are sampling devices that are less commonly used than nets. They occurred in 16% of the analyzed studies (Fig. 3a, Table 1). These systems can consist of different kinds of pumps (Lusher et al. 2014; Setälä et al. 2016; Zobkov et al. 2019) and allow for sampling a large volume of seawater. Seawater intake systems of the vessel can be used for this purpose (Morgana et al. 2018). Pumps can be lowered from the vessel using a winch sideways or towards the stern of the ship (Ng and Obbard 2006; Setälä et al. 2016). There is no standardization for sampling time with pumping systems and they can work for several hours straight along the same transect (Lenz et al. 2015) or for a few minutes or hours in different sampling stations (Desforges et al. 2014; Zobkov et al. 2019). Pumping systems allow for sampling the sub-superficial layer of the water column from surface to 6-m depth or down to a depth of 100 m (Zhao et al. 2015; Morgana et al. 2018; Zobkov et al. 2019). When sampling is performed continuously, the vessel speed must be kept at 1–12 knots (Enders et al. 2015; Setälä et al. 2016). The dimension of the sampled MP depends on the filters/sieves of the pumping systems, which allow for selecting plastic of the size of interest. First, there is a filter with a 5-mm mesh size used to remove bigger plastic particles, then a second filter or a series of sieves with a different mesh size that divides MPs into size classes (Desforges et al. 2014; Cai et al. 2018).

At the end of the sampling, MPs can be directly analyzed on filters, whereas sieves need to be rinsed with decontaminated water (e.g., Milli-Q water) and MPs collected in a glass jar and preserved until laboratory analysis. Pumping systems can be equipped with a flow meter to determine the rate of filtered water, which can be used to calculate MP abundance and/or mass for volume unit (Cincinelli et al. 2017).

### Discrete sampling devices

Discrete sampling devices are used to sample water at specific depths. They were employed in only 19% of the analyzed studies (Fig. 3a, Table 1). Discrete sampling devices such as Niskin bottle (Bagaev et al. 2018), rosette (Dai et al. 2018; Kanhai et al. 2018), IWS (Tamminga et al. 2018) are common in water sampling for different purposes, such as analysis of total suspended solids. For this reason, they are widely known in the literature. Moreover, buckets, bottles, and steel samplers are also used (Dubaish and Liebezeit 2013; Khalik et al. 2018; Zhu et al. 2019). These devices are lowered manually or through a winch from the vessel down to the sampling depth (Bagaev et al. 2018).

At the end of sampling with discrete devices, the sample is transferred to a jar and the inside of the device is rinsed with decontaminated water (e.g., microfiltered water) to collect MP particles that remain attached. The sample is preserved until laboratory analysis. In another way, discrete samples can be filtered (Dai et al. 2018) or sieved (Kanhai et al. 2018) directly on board.

### Sampling devices for the surface microlayer

Sampling devices for the sea surface microlayer can collect seawater within the first micrometers of the water column, and only 4% of the analyzed studies used them (Fig. 3a, Table 1). They are stainless-steel sieves with 2-mm mesh size and a rotating drum sampler that are placed on the surface of the water (Ng and Obbard 2006; Song et al. 2015).

During sampling, the sieve is placed in contact with the seawater surface, which is retained within the mesh of the sieve due to surface tension and is collected in a stainless-steel plate. This procedure is repeated for a specific amount of time, e.g., 100 times (Song et al. 2015). Water collected in the plate is then transferred to a jar and preserved until laboratory analysis. A rotating drum sampler is placed on the seawater surface at the beginning of sampling. It consists of a glass cylinder that rotates while partially submerged, and it has a hydrophilic clean surface, which, using capillary force, can collect samples from the surface microlayer (from 1 to 1000 μm depths; Ng and Obbard 2006; Hidalgo-Ruz et al. 2012).

### MP sample preservation methods

Once the sampling process ends, different sample preservation methods can be used. To preserve the biological component of samples for laboratory analysis, preservation methods mainly involve the use of 4% formalin, which is added to the sample before it is sealed for transfer to the laboratory (van der Hal et al. 2017; Figueiredo and Vianna 2018). If MPs are the only parameters of interest in a study, ethanol or sample refrigeration can be used as a preservative (Castillo et al. 2016; Viršek et al. 2016).

## Laboratory methodologies for MP analysis

**Fig. 4:**
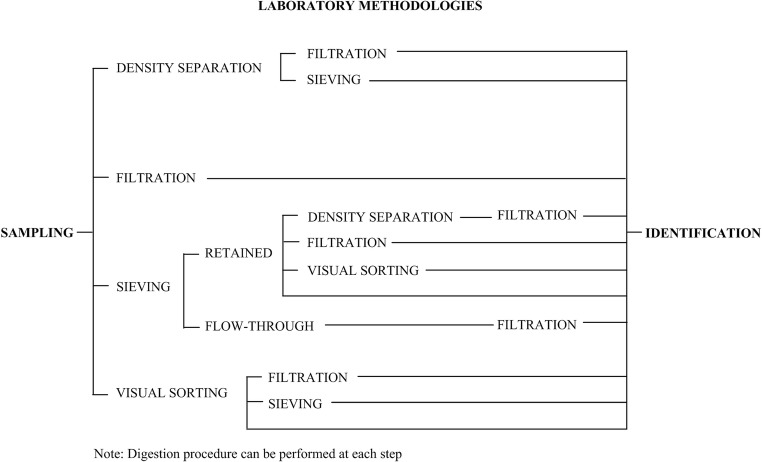
Scheme of different steps of laboratory methodologies applied to separate microplastics from seawater in the studies analyzed in the present review

MP samples must undergo one or more separation processes to isolate the MP from seawater. The MP can be directly identified and classified, without separation processes, only when it remains already collected on a filter after sampling. Separation methods can be classified into the following: density separation, filtration, and sieving (Fig. 4; Hidalgo-Ruz et al. 2012). Visual sorting of samples can be performed prior to filtration or sieving to remove MPs with sizes larger than 5 mm, which are visible with the naked eye (Maes et al. 2017), or it can also be performed after sieving by removing the MPs retained on the sieve (Fig. 4) (Viršek et al. 2016) (Figure 4). Moreover, different kinds of sample digestion (acidic, enzymatic, alkaline, and oxidative) can be performed to separate MPs from biological matter (Miller et al. 2017).

### Density separation

Density separation removes MPs from the rest of the sample, exploiting the floating properties of MPs in different solutions which need to be denser than MPs. Supernatant-containing MPs are collected and filtered (Barrows et al. 2017) (Fig. 4). MPs on filters are finally identified and classified. Sodium chloride (NaCl) solutions are the most used (Galgani et al. 2013). Other density separation methods can be applied to obtain a denser solution and to float even the densest MPs, and they involve the use of zinc chloride (ZnCl_2_) (Syakti et al. 2018), sodium iodide (NaI) solution (Saliu et al. 2018), or surfactants such as sodium lauryl sulfate (SLS) (Lenz et al. 2015).

### Filtration

Gravity filtration or vacuum filtration can be performed. Different kinds of filters can be used to separate MPs from seawater, including polycarbonate (Norén 2007), polyamide (Enders et al. 2015), nylon (Tang et al. 2018), glass fiber (Pan et al. 2019), cellulose acetate (Castro et al. 2016), mixed cellulose ester (Desforges et al. 2014), cellulose nitrate (Dubaish and Liebezeit 2013), and Anopore inorganic membrane (Anodisc) filters (Saliu et al. 2018). Filter diameters of 45 mm (Norén 2007) and 47 mm (Zhu et al. 2019) are the most frequently used. The pore sizes of the filters used for MP samples range from 0.2 μm (Syakti et al. 2018) to 300 μm (Setälä et al. 2016). Gridded filters with clearly defined grid lines are used to facilitate MP quantification (MERI 2017).

Samples can be directly filtered (no pre-treatment is necessary with bulk seawater samples) or they can undergo previous treatments step like sieving, which help retain MP particles (Fig. 4). Then, decontaminated water (e.g., micro-filtered water) is used to remove MPs from the sieve (Lusher et al. 2014). In some cases, MP particles retained by the sieve are directly subjected to visual analysis, while MPs contained in the flow passing through the sieve are filtered (Erni-Cassola et al. 2017) (Fig. 4). Otherwise, the original MP sample can be left to settle to obtain the deposition of the inorganic and biological material on the bottom. Then, the supernatant is filtered, whereas the precipitate is sieved (Castro et al. 2016) (Fig. 4).

In the final phase of the filtration procedure, filters can be rinsed with pure water, such as ultrapure water, to avoid the formation of salt crystals on the dry filters (Palatinus et al. 2015). Filters are preserved in previously cleaned glass Petri dishes, and remaining solutions can be removed by an oven or a drier (40–70 °C; Hall et al. 2015) or at room temperature (Barrows et al. 2017). The temperature must be carefully chosen since some plastics melt at temperatures lower than 70 °C; however, most of the plastics melt at temperatures higher than 100 °C (Sigma Aldrich 2019). Dried filters are weighed to determine the MP mass (Song et al. 2015) or they can undergo a digestion procedure (Abayomi et al. 2017).

### Sieving

Sieving allows for the separation of MPs from seawater using one or more metal sieves. It can be performed with only one sieve or with a series of sieves. The number of sieves used, and their mesh sizes depend on the sieving goal, such as selection of a specific MP size range, removal of a specific fraction of MPs (e.g., plastic particles with sizes greater than 5 mm), or partition of the MPs into size classes (Masura et al. 2015).

Sieving can be carried out directly on the vessel (Desforges et al. 2014) or in the laboratory, and it can be performed on the MP sample once it has been separated from seawater through different methods, such as visual sorting, density separation, or digestion methods (Lima et al. 2014; Masura et al. 2015; Suaria et al. 2016; Kroon et al. 2018) (Fig. 4).

To start a sieving procedure, MP samples are poured onto a sieve or a series of sieves and then rinsed with decontaminated water (e.g., Milli-Q water). When the sieving process is concluded, the sieve is rinsed accurately with pre-filtered water to collect all MP particles (Maes et al. 2017). MPs on each sieve can be placed or rinsed into Petri dishes or glass jars (Dubaish and Liebezeit 2013; Desforges et al. 2014) or they can be filtered using rinse water (Lusher et al. 2014) (Fig. 4). MPs retained by the sieve undergo visual sorting, whereas those that flow through the sieve can undergo filtration (Kang et al. 2015; Erni-Cassola et al. 2017) (Fig. 4). MPs placed on filters or into Petri dishes and glass jars can be dried in an oven or a drier (50–65 °C or at room temperature; Collignon et al. 2012; Palatinus et al. 2015) and then weighted.

### Digestion methods

Digestion methods are separation techniques that isolate the MP from the organic matter (Miller et al. 2017). In fact, digestion methods are used to degrade the organic part of the sample, whereas MPs are not removed. There are different types of digestion, including acidic (Zobkov et al. 2019), enzymatic (Saliu et al. 2018), alkaline (Zhu et al. 2019), and oxidative (Pan et al. 2019), and they can also be used in combination (Tamminga et al. 2018). The use of different digestion methods allows for the degradation of a wider range of compounds. Digestion processes take place after MP separation from seawater, and they are usually coupled with density separation (Masura et al. 2015).

Substances that are commonly used during the digestion procedure are hydrogen peroxide (H_2_O_2_), with different volumes (30% volumes most used; Dai et al. 2018), H_2_O_2_ combined with a catalyst such as ferrous sulfate (FeSO_4_) (Masura et al. 2015), hydrochloric acid (HCl) (Zobkov et al. 2019), hydrofluoric acid (HF) (Dubaish and Liebezeit 2013), sodium hydroxide (NaOH) (Cole et al. 2014) and/or potassium hydroxide (KOH) (Beer et al. 2018), sodium hypochlorite (NaClO) (Beer et al. 2018), and different enzymes, of which the most common is proteinase K (Saliu et al. 2018). Substances used for digestion of organic matter can be used in combination, such as 30% H_2_O_2_, followed by treatment of the sample with 40% HF (Dubaish and Liebezeit 2013). To remove organic matter, wet peroxide oxidation (WPO) can also be used (Sutton et al. 2016). After sample digestion, MP samples can undergo other separation processes (e.g., filtration) or they can be identified and classified. Acid digestion using a strong acid, such as HCl or HF, can compromise the structural integrity of the MP (Miller et al. 2017), therefore their use is not recommended.

## MP identification techniques

MP identification techniques can be divided into visual and analytical techniques. Visual identification provides for MP classification and identification with the naked eye or using a microscope. Analytical techniques (spectroscopy, gas-chromatography, etc.) require the use of different laboratory equipment generally used in analytical chemistry. These techniques allow for the identification of MP polymer.

### Visual identification techniques

When studies on MPs started, visual identification techniques are primarily used to identify and classify MPs. They consist of the observation of MPs preserved on a filter (Song et al. 2015) or in Petri dishes (Palatinus et al. 2015) or jars (Reisser et al. 2013) using the naked eye or a microscope. For this purpose, different microscopes can be used, including fluorescence (Cai et al. 2018), dissection (Sagawa et al. 2018), optical (Bagaev et al. 2018), electron (Leslie et al. 2011), stereo (Zobkov et al. 2019), binocular (de Lucia et al. 2014), inverted (Gorokhova 2015), and vertical (Setälä et al. 2016) microscopes. Microscope magnification used for MP identification varies from × 4.5 (MERI 2017) to × 400 (Norén 2007).

A scanning electron microscope (SEM) can also be used. Due to its high resolution, it allows for the most precise MP identification, even if it is not possible to distinguish color (Sagawa et al. 2018). Microscopes may be equipped with a camera, and therefore, MPs can also be photographed and subsequently analyzed (Sun et al. 2018). To facilitate visual analysis, especially under the microscope, samples that undergo filtration are generally placed on gridded filters (MERI 2017).

Three criteria have been established for MP recognition, which allows for discerning MPs from other materials, in particular, biological matter, as follows: MP particles must not have visible cellular or organic structures; fibers should be equally thick throughout their entire length; and particles should exhibit clear and homogeneous color throughout (Norén 2007; Hidalgo-Ruz et al. 2012; MERI 2017). If these criteria are followed, particles can be defined as plastic (Hidalgo-Ruz et al. 2012).

Furthermore, a melting test and hot needle test can be performed to assess if the observed particles are effectively made of plastic (Enders et al. 2015; Tunçer et al. 2018). These tests consist of burning particles and the burned particle is defined as plastic if it melts or wrinkles (MERI 2017). Specifically, the hot needle test requires the use of a needle previously heated to high temperatures (Gorokhova 2015). These tests compromise or cause the loss of the analyzed particle, and for this reason, they are performed only on particles of uncertain nature.

### Analytical identification techniques

The application of analytical techniques comes after visual sorting, and it requires different instruments to determine if particles are made of plastic and for MP polymer identification. The most common instrument used is Fourier-transform infrared (FTIR) spectroscopy (Table 3); MPs can be analyzed individually or on a filter depending on their dimensions (Ng and Obbard 2006; Gallagher et al. 2016). For spectroscopy techniques, instruments can be coupled with a microscope for identification of MPs of very small dimensions (< 1 mm). These include micro-FTIR (Frias et al. 2014; Sun et al. 2018), micro-attenuated total reflection FTIR (ATR-FTIR) (Palatinus et al. 2015), focal plane array (FPA) detector-based micro-Fourier-transform infrared (FPA-micro-FTIR), and micro-Raman (Enders et al. 2015; Erni-Cassola et al. 2017) (Table 3). If analytical identification techniques that exploit the thermal stability properties of MPs are applied, destruction of the sample occurs, as when using differential scanning calorimetry (DSC) (Tunçer et al. 2018).

**Table 3:** Analytical identification techniques of microplastics used in the studies analyzed in the present review. To simplify the table, references are here indicated with a number and the corresponding complete list is presented in the Online Resource 1

Microplastics analytical identification techniques	Number of studies	References (Online Resource 1)
Fourier-transform infrared spectroscopy (FTIR)	12	[1], [4], [9], [13], [29], [34], [35], [37], [43], [51], [52], [58]
Micro-Fourier transform infrared spectroscopy (Micro-FTIR)	15	[9], [12], [16], [25], [27], [31], [32], [36], [37], [46], [56], [60], [65] [66], [73]
Attenuated total reflection Fourier transform infrared spectroscopy (ATR-FTIR)	15	[7], [8], [10], [11], [33], [38], [39], [45], [53], [59], [62], [63], [67], [68], [70]
Micro-attenuated total reflection Fourier transform infrared spectroscopy (Micro ATR-FTIR)	4	[53], [55], [57], [70]
Fourier Transform Near-Infrared spectroscopy (FT-NIR)	1	[1]
Raman spectroscopy	2	[7], [42]
Micro-Raman spectroscopy	6	[21], [23], [40], [49], [71], [74]
Semi-automated micro-Raman spectroscopy	1	[26]
Near Infrared Spectroscopy (NIR)	1	[28]
Differential scanning calorimetry (DSC)	1	[68]

All analytical techniques result in a spectrum related to each analyzed particle. To identify which polymer the particles are made of, each spectrum is compared with a reference. Therefore, these techniques allow for unequivocally determining if particles are made of plastic. To facilitate MP identification, it is possible to use artificial colors, and Nile Red is the fluorescent color most commonly used (Desforges et al. 2014; Tamminga et al. 2018).

**Fig. 5:**
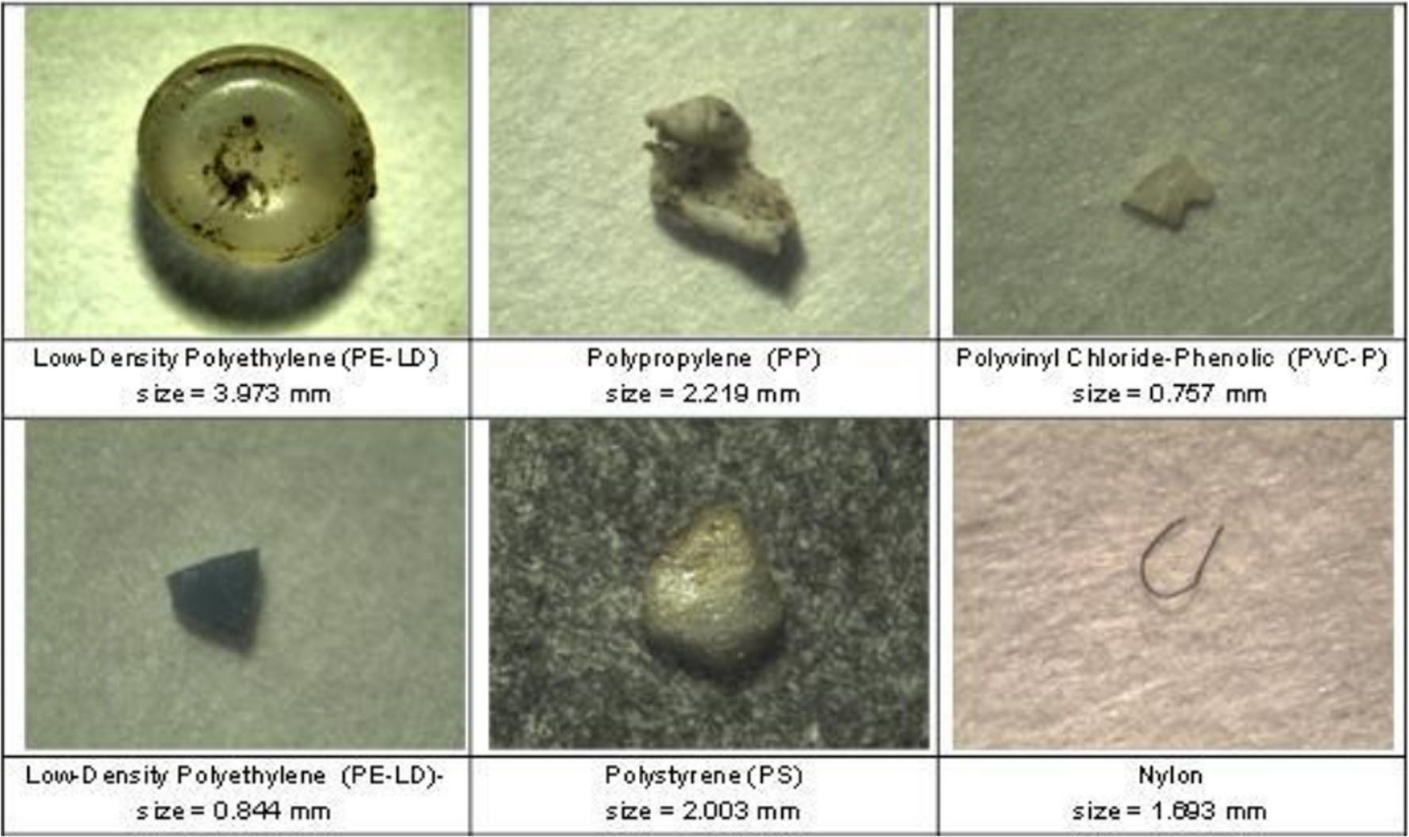
Example of microplastic particles and their polymer type and their size (Galgani et al. 2013)

Analytical techniques are time-consuming because MPs are analyzed one by one, and they are expensive (Frère et al. 2016). For these reasons, for 17 studies analyzed, only part of the MP was analyzed with analytical techniques; this subset must be representative of all MPs collected during sampling, extending the result of the analytical identification to the bulk sample. Some examples of MP particles analyzed to determine their polymeric composition are shown in Fig. 5.

## How to report MP results

### MP classification

MP classification is based on different morphological characteristics, such as the type (plastic fragments, pellets, filaments, plastic films, foamed plastics, granules, and styrofoam), shape (cylindrical, disks, flat, ovoid, spheruloids, rounded, subrounded, subangular, angular, irregular, elongated, degraded, rough, and broken edges), color (transparent, crystalline, white, clear-white-cream, red, orange, blue, opaque, black, gray, brown, green, pink, tan, yellow), size, and type of polymer (Galgani et al. 2013).

Different studies used MP classification methods that are slightly different from each other and for this reason, the results of these studies are not comparable.

### Abundance and concentration of MPs

Results from MP studies can be reported as abundance or concentration of MP in seawater (Table 4). MP abundance is expressed as surface units (number of MP km^−2^, number of MP m^−2^) when MPs are collected from the surface layer of the water column. Moreover, MP abundance can be reported as units of volume. The number of MP m^−3^ is the most used, but the number of MP L^−1^ is also used (Table 4). MP concentration can also be estimated from its dry weight and then reported as unit area or units of volume (g or mg of MP km^−2^, g or mg of MP m^−3^) (Suaria et al. 2016; Baini et al. 2018; Erni-Cassola et al. 2017; Syakti et al. 2018).

**Table 4:** Microplastics (MP) reporting units in the reviewed studies. The number of studies is equal to 78 instead of the 74 reviewed because some studies did not specify the microplastics reporting unit and others used more than one type of unit. To simplify the table, references are here indicated with a number and the corresponding complete list is presented in Online Resource 1

Microplastics reporting unit	Number of studies (*n* = 78)	References (Online Resource 1)
Number of microplastics per square kilometer (MP km^−2^)	16	[1], [4], [5], [22], [24], [28], [32], [42], [44], [49], [51], [61], [64], [68], [69], [70]
Number of microplastics per square meter (MP m^−2^)	4	[14], [15], [52], [59]
Number of microplastics per cubic meter (MP m^−3^)	40	[2], [4], [6], [7], [9], [10], [11], [12], [13], [17], [18], [21], [25], [27], [28], [30], [34], [25], [36], [37], [41], [42], [43], [45], [47], [53], [54], [59], [60], [62], [63], [64], [65], [66], [67], [68], [69], [71], [72], [74]
Number of microplastics per liter (MP L^−1^)	12	[3], [5], [16], [19], [31], [33], [38], [50], [56], [57], [64], [73]
Grams of microplastics per square kilometers (g MP km^−2^)	2	[4], [59]
Grams of microplastics per cubic meter (g MP m^−3^)	1	[7]
Milligrams of microplastics per cubic meter (mg MP m^−3^)	2	[59], [62]
Milligrams of microplastics per square kilometers (mg MP km^−2^)	1	[24]

## How to avoid sample contamination

Studies on MP content in seawater are particularly affected by air contamination of the sample. Potential sources of contamination during sampling include clothes, all equipment that come into contact which sampling devices such as the paint of the vessel, all kinds of plastic material on the vessel, and devices used to move samples from collecting jars to storage jars (Bagaev et al. 2018; Baini et al. 2018). To avoid contamination as much as possible, it is strongly recommended to wear non-plastic clothes, such as a cotton lab coat and nitrile or latex gloves (Kanhai et al. 2018; Morgana et al. 2018). Alternatively, it is possible to wear clothes and gloves of a color that can easily be recognized or to record materials and colors of the equipment in a way to remove them during visual sorting of the sampled MP (Bagaev et al. 2018).

In the same way, it is preferred that sampling and lab instruments be made of non-plastic materials, such as glass (Bimali Koongolla et al. 2018). If plastic items are used, they should be disposable (Erni-Cassola et al. 2017). Otherwise, it is necessary to check possible contamination from the item, analyzing the amount of pre-filtered and preferably distilled water that was used to pre-wash it (Kanhai et al. 2018). Pre-washing of sampling devices and lab equipment is mandatory, using distilled/pre-filtered water (Zhu et al. 2019), acetone (Beer et al. 2018), 70% ethanol (Kroon et al. 2018), or an acid (Cole et al. 2014). Prior to sampling, checking for possible contamination from the sieves and filters of the sampling device and water used to pre-rinsed them can be useful (Sutton et al. 2016). To avoid contamination from liquids used during the laboratory procedures, pre-filtration is necessary (Qu et al. 2018).

Minimizing air exposure, both during sampling and in the laboratory, is a minimum preventive measure (Zhu et al. 2019); analysis of the MP content of the seawater sample should always occur under a laminar flow hood (Tamminga et al. 2018). In addition, it is possible to investigate possible contamination in the laboratory by analyzing a sample of air sucked through a filter (Zhao et al. 2015). A good habit that is extremely useful for assessing sample contamination is placing a blank filter in a Petri dish close to the area where sampling and laboratory protocols occur, which must be left open during the entire process. Subsequently, the blank filter must go through the same analysis steps of the sample to compare them and eventually remove MPs found in the blank from the sample results (Lusher et al. 2014; Barrows et al. 2017). Another type of blank is the analysis of negative control (e.g., distilled water): the eventual MP content must be compared with the results from the analysis of the samples (Song et al. 2015; Cai et al. 2018).

## Discussion and conclusions

Sampling strategies, laboratory analyses, and identification techniques of MPs are numerous, and there is a lack of procedure standardization although a great effort has been underway in this direction in recent years (Gago et al. 2018; GESAMP 2019). The use of different methods makes it difficult to compare different study results. Moreover, sampling and laboratory analyses of MPs are performed with devices and equipment that are already used to investigate other parameters, and therefore in some cases, they are not appropriate for the study of microplastics. For example, because some sampling devices are made of plastic material, water samples can be contaminated by them, and a control sample is always necessary. After reviewing 74 papers, the authors present the following suggestions regarding different aspects of researching MPs in seawater samples from a port environment.

### Sampling devices

A sampling device determines the lower limit in size of the MP collected. A mesh size that is too small (e.g., 10 μm) is hard to apply and can easily get clogged, mostly in a marine environment where suspended particles can be very concentrated such as ports, or during phytoplankton blooms.

#### Nets

Nets are strongly recommended for MP sampling, especially from surface seawater in large basin or channels. Mesh sizes of 200, 330, and 333 μm are the most commonly used, but, if employed alone, the study lacks the evaluation of MPs smaller than the net mesh size. Despite this, nets are very useful devices because of the following characteristics: fast collection of MPs from a large amount of water in a wide area; not very expensive; no electricity needed; no highly specialized expertise required. However, because of net length and the fact that net must be kept away from the boat, the horizontal use of a net for MP sampling within a port basin can be difficult due to limited spaces, presence of piers, naval traffic, and presence of obstacles (ships, anchors, moorings, and ropes) that typically characterize port basins. Net towing can therefore be suitable only in long channels present in ports. In the same way, the vertical use of net can be hindered by the low depths that often characterize water column inside port and the presence of obstacles (debris, moorings, etc.) in the water column near piers.

#### Pumping systems

Pumping systems can be coupled with a sieve which directly separate MPs from a larger amount of water. They can be easily used in a port environment because they do not have a large clutter and, therefore, allow sampling in restricted areas, such as between piers and vessels. However, they are more expensive, electricity-consuming, and difficult to clean in between sampling.

#### Discrete sampling devices

Discrete sampling devices allow sampling even in restricted areas and are therefore suitable for sampling in ports; moreover, they are easier to apply than pumping systems because they are not expensive, not electricity-consuming, and easy to check for contamination. However, these are only able to collect MPs without any pre-selection in terms of size, thus the results depend only on the laboratory methodologies used (e.g., mesh size of sieves or pore size of filters).

#### Surface microlayer sampling devices

Both the sieve and rotating drum sampler must be used with calm sea to obtain samples of good quality. Since MPs can be found at different depths, from the surface down to the bottom (Dai et al. 2018), the sole sampling of the surface microlayer provides an underestimation of the MPs in a study area. In addition, given the small area that can be sampled using these devices, the operations must be repeated numerous times to obtain a sufficiently representative area coverage of the study area. However, these devices allow sampling in even restricted areas and can therefore be useful in port areas with numerous obstructions.

All these considerations are in accordance with the “Guidelines for the monitoring and assessment of plastic litter in the ocean”, which do not recommend any device, relating the choice to the characteristics of the sampling area, the instrumentation availability, and the aim of the study (GESAMP 2019). However, if the purpose of the research is the characterization of the MP occurrence in a specific area such as port, we would recommend using more than just one sampling device. For example, MPs from surface seawater may be collected using a net along channel (if present inside the port), while surface water between piers may be collected with a pumping system or a Niskin bottle; those last devices can also be used in parallel with nets to avoid the loss of MPs smaller than the mesh size.

### Laboratory procedures and contamination control

The use of hyper-saturated NaCl saline solution is the most convenient density separation method, due to its low cost and easy availability compared with the other solutions. Solutions of NaI or ZnCl_2_ are not recommended since they are dangerous to handle and very expensive.

Filtration is the most reasonable procedure to separate MPs from seawater, and almost every study applies it. In fact, comparing filtering with sieving, the first can be applied with a smaller pore than a sieve, collecting a wider size range of MPs. In addition, filtering is less subject to contamination because filters are disposable, while sieves need to be carefully rinsed in between different samples. However, the presence of a blank filter, which must be left open close to the working area and whose use is very common in between the reviewed studies (e.g., Baini et al. 2018; Kanhai et al. 2018), is strictly recommended and should be assessed as mandatory during the sample filtering procedure. This type of blank seems to be more useful than using a procedural control of filtered distilled water, as air contamination is likely to happen. It is necessary to choose the materials for the filter prior to beginning the study, which depends on the type of analysis performed. In fact, if an analytical identification technique is used, it is mandatory to avoid the use of a filter made of plastic material, which may interfere with the analysis. Digestion of organic matter is a crucial step in the analysis of MPs in water samples taken in the port environment, as very often they are very charged with organic matter that would make it difficult to recognize MPs and disturb polymer analysis.

### MP identification

About analytical identification, the visual sorting technique cannot be performed alone, since it is subject to the interpretation of the operator who conducts the analysis and may lead to the underestimation of MPs due to difficulty in observing the smallest ones (< 1 mm) with the naked eye and MPs with micrometric dimensions with the microscope (Hidalgo-Ruz et al. 2012). To reduce the risk of underestimation, the visual sorting of a sample must be performed by more than one trained operator (Gajšt et al. 2016). Analytical techniques are the only techniques that can assess whether particles are made of plastics and demonstrate how a large part of particles visually defined as plastic is actually made of other materials (e.g., organic matter, cotton fibers, and glass) (Frère et al. 2016). However, analytical techniques are expensive and time-consuming; therefore, in most cases, they are not performed for all sampled MP particles. It is mandatory to carry out analytical analysis on at least a subset of samples to validate results obtained by visual analysis, and since MPs are very tiny, it is recommended that a microscope be coupled with instruments such as Raman and FTIR to perform a high-quality analysis.

### Result reporting

Finally, it is not to be underestimated how MP concentrations are reported. In fact, data presenting different units of measurement cannot be compared. Already in 2013, the first “Guidance on monitoring of marine litter in European Seas” (Galgani et al. 2013) pointed out MP m^−3^ as a useful unit of measurement, and we completely agree with this solution, which should be part of a standard protocol. In addition, we believe that data on seawater surface samples should be presented using MP m^−2^ since the MP m^−3^ unit allows for comparing results with other studies or matrices, but the MP m^−2^ unit is more useful to realistically characterize a wide but thin area, such as the seawater surface.

Since a standard protocol for MP investigation in seawater does not exist yet, this study can be a useful tool for taking advantage of both successes and failures of the previous studies; in addition, the authors suggest the use of this review for choosing the best techniques to sample and analyze MPs in such a particular environment as ports.

#### Fundign information

The present study was funded by the European Interreg Italy-France Maritime 2014-2020 Project “SPlasH! - Stop alle Plastiche in H_2_O” (CUP D31I18000620007).

## Electronic supplementary material

ESM 1 (PDF 145 kb)

## References

[CR1] Abayomi OA, Range P, Al-Ghouti MA, Obbard JP, Almeer SH, Ben-Hamadou R (2017). Microplastics in coastal environments of the Arabian Gulf. Mar Pollut Bull.

[CR2] Bagaev A, Khatmullina L, Chubarenko I (2018). Anthropogenic microlitter in the Baltic Sea water column. Mar Pollut Bull.

[CR3] Baini M, Fossi MC, Galli M, Caliani I, Campani T, Finoia G, Panti C (2018). Abundance and characterization of microplastics in the coastal waters of Tuscany (Italy): the application of the MSFD monitoring protocol in the Mediterraneand Sea. Mar Pollut Bull.

[CR4] Barnes DKA, Galgani F, Thompson RC, Barlaz M (2009). Accumulation and fragmentation of plastic debris in global environments. Philos T R Soc.

[CR5] Barrows APW, Neumann CA, Berger ML, Shaw SD (2017). Grab *vs.* neuston tow net: a microplastic sampling performance comparison and possible advances in the field. Anal Methods-UK.

[CR6] Beer S, Garm A, Huwer B, Dierking J, Nielsen TG (2018). No increase in marine microplastic concentration over the last three decades – a case study from the Baltic Sea. Sci Total Environ.

[CR7] Bimali Koongolla J, Andrady AL, Terney Pradeep Kumara PB, Gangabadage CS (2018). Evidence of microplastics pollution in coastal beaches and waters in southern Sri Lanka. Mar Pollut Bull.

[CR8] Cai M, He H, Liu M, Li S, Tang G, Wang W, Huang P, Wei G, Lin Y, Chen B, Hu J, Cen Z (2018). Lost but can’t be neglected: huge quantities of small microplastics hide in the South China Sea. Sci Total Environ.

[CR9] Castillo AB, Al-Maslamani I, Obbard JP (2016). Prevalence of microplastics in the marine waters of Qatar. Mar Pollut Bull.

[CR10] Castro RO, Silva ML, Marques MRC, de Araújo FV (2016). Evaluation of microplastics in Jurujuba Cove, Niterói, RJ, Brazil, an area of mussels farming. Mar Pollut Bull.

[CR11] Cincinelli A, Scopetani C, Chelazzi D, Lombardini E, Martellini T, Katsoyiannis A, Fossi MC, Corsolini S (2017). Microplastic in the surface waters of Ross Sea (Antarctica): occurrence, distribution and characterization by FTIR. Chemosphere.

[CR12] Cole M, Webb H, Lindeque PK, Fileman ES, Halsband C, Galloway TS (2014). Isolation of microplastics in biota-rich seawater samples and marine organisms. Sci Rep-UK.

[CR13] Collignon A, Hecq JH, Galgani F, Voisin P, Collard F, Goffart A (2012). Neustonic microplastic and zooplankton in the North Western Mediterranean Sea. Mar Pollut Bull.

[CR14] Collignon A, Hecq J, Galgani F, Collard F, Goffart A (2014). Annual variation in neustonic micro- and meso-plastic particles and zooplankton in the Bay of Calvi (Mediterranean-Corsica). Mar Pollut Bull.

[CR15] Cózar A, Echevarría F, Gonzalez-Gordillo JI, Irigoien X, Úbeda B, Hernández-Leon S, Palma AT, Navarro S, García-de-Lomas J, Ruiz A, Fernández-de-Puelles ML, Duarte CM (2014). Plastic debris in the open ocean. P Natl A Sci India.

[CR16] Dai Z, Zhang H, Zhou Q, Tian Y, Chen T, Tu C, Fu C, Luo Y (2018). Occurrence of microplastics in the water column and sediment in an inland sea affected by intensive anthropogenic activities. Environ Pollut.

[CR17] de Lucia GA, Caliani I, Marra S, Camedda A, Coppa S, Alcaro L, Campani T, Giannetti M, Coppola D, Cicero AM, Panti C, Baini M, Guerranti C, Marsili L, Massaro G, Fossi MC, Matiddi M (2014). Amount and distribution of neustonic micro-plastic off the western Sardinian coast (Central-Western Mediterranean Sea). Mar Environ Res.

[CR18] Derraik JGB (2002). The pollution of the marine environment by plastic debris: a review. Mar Pollut Bull.

[CR19] Desforges JPW, Galbraith M, Dangerfield N, Ross PS (2014). Widespread distribution of microplastics in subsurface seawater in the NE Pacific Ocean. Mar Pollut Bull.

[CR20] Dubaish F, Liebezeit G (2013). Suspended microplastics and black carbon particles in the jade system, southern North Sea. Water Air Soil Pollut.

[CR21] Duis K, Coors A (2016). Microplastics in the aquatic and terrestrial environment: sources (whit a specific focus on personal care products), fate and effects. Environ Sci Eur.

[CR22] Enders K, Lenz R, Stedmon CA, Nielsen TG (2015). Abundance, size and polymer composition of marine microplastics ≥ 10 μm in the Atlantic Ocean and their modelled vertical distribution. Mar Pollut Bull.

[CR23] Eriksen M, Liboiron M, Kiessling T, Charron L, Alling A, Lebreton L, Richards H, Roth B, Ory NC, Hidalgo-Ruz V, Meerhoff E, Box C, Cummins A, Thiel M (2018). Microplastic sampling with the AVANI trawl compared to two neuston trawls in the Bay of Bengal and South Pacific. Mar Pollut Bull.

[CR24] Erni-Cassola G, Gibson MI, Thompson RC, Christie-Oleza JA (2017). Lost, but found with Nile red: a novel method for detecting and quantifying small microplastics (1 mm to 20 μm) in environmental samples. Environ Sci Technol.

[CR25] European Commision (2017). Commission decision (EU) 2017/848 of 17 May 2017 laying down criteria and methodological standards on good environmental status of marine waters and specifications and standardised methods for monitoring and assessment, and repealing decision 2010/477/EU. Off J Eur Union L.

[CR26] European Commission (2008). Directive 2008/56/EC of the European Parliament and of the council establishing a framework for community action in the field of marine environmental policy (marine strategy framework directive). Brussels: Off J Eur Union.

[CR27] Figueiredo GM, Vianna TMP (2018). Suspended microplastics in a highly polluted bay: abundance, size, and availability for mesozooplankton. Mar Pollut Bull.

[CR28] Frère L, Paul-Pont I, Moreau J, Soudant P, Lambert C, Huvet A, Rinnert E (2016). A semi-automated Raman micro-spectroscopy method for morphological and chemical characterizations of microplastic litter. Mar Pollut Bull.

[CR29] Frias JPGL, Otero V, Sobral P (2014). Evidence of microplastics in samples of zooplankton from Portuguese coastal waters. Mar Environ Res.

[CR30] Gago J, Galgani F, Maes T, Thompson RC (2016). Microplastics in seawater: recommendations from the marine strategy framework directive implementation process. Front Mar Sci.

[CR31] Gago J, Carretero O, Filgueiras AV, Viñas L (2018). Synthetic microfibers in the marine environment: a review on their occurence in seawater and sediments. Mar Pollut Bull.

[CR32] Gajšt T, Bizjak T, Palatinus A, Liubartseva S, Kržan A (2016). Sea surface microplastics in Slovenian part of the northern Adriatic. Mar Pollut Bull.

[CR33] Galgani F, Hanke G, Werner S, Oosterbaan L, Nilsson P, Fleet D, Kinsey S, Thompson R, Palatinus A, Van Franeker JA, Vlachogianni T, Scoullos M, Veiga JM, Matiddi M, Alcaro L, Maes T, Korpinen S, Budziak A, Leslie H, Gago J, Liebezeit G (2013) Guidance on monitoring of marine litter in European Seas. Report EUR 26113 EN – Joint Research Centre – Institute for Environment and Sustainability, pp. 128

[CR34] Gallagher A, Rees A, Rowe R, Stevens J, Wright P (2016). Microplastics in the Solent estuarine complex, UK: an initial assessment. Mar Pollut Bull.

[CR35] GESAMP (2019) Guidelines or the monitoring and assessment of plastic litter and microplastics in the ocean. In: Kershaw PJ, Turra A, Galgani F (eds). (IMO/FAO/UNESCO-IOC/UNIDO/WMO/IAEA/UN/UNEP/UNDP/ISA Joint Group of Experts on the Scientific Aspects of Marine Environmental Protection). Rep. Stud. GESAMP no. 99, pp. 130

[CR36] Gorokhova E (2015). Screening for microplastic particles in plankton samples: how to integrate marine litter assessment into existing monitoring programs?. Mar Pollut Bull.

[CR37] Green DS, Kregting L, Boots B, Blockley DJ, Brickle P, da Costa M, Crowley Q (2018). A comparison of sampling methods for seawater microplastics and a first report of the microplastic litter in coastal waters of Ascension and Falkland Islands. Mar Pollut Bull.

[CR38] Hall NM, Berry KLE, Rintoul L, Hoogenboom MO (2015). Microplastic ingestion by scleractinian corals. Mar Biol.

[CR39] Hidalgo-Ruz V, Gutow L, Thompson RC, Thiel M (2012). Microplastics in the marine environment: a review of the methods used for identification and quantification. Environ Sci Technol.

[CR40] Ivar do Sul JA, Costa MF (2014). The present and future of microplastic pollution in the marine environment. Environ Pollut.

[CR41] Kang JH, Kwon OY, Lee KW, Song YK, Shim WJ (2015). Marine neustonic microplastics around the southeastern coast of Korea. Mar Pollut Bull.

[CR42] Kanhai LDK, Gårdfeldt K, Lyashevska O, Hassellöv M, Thompson RC, O’Connor I (2018). Microplastics in sub-surface waters of the Arctic Central Basin. Mar Pollut Bull.

[CR43] Karbalaei S, Hanachi P, Walker TR, Cole M (2018). Occurrence, sources, human health impacts and mitigation of microplastic pollution. Environ Sci Pollut Res.

[CR44] Khalik WMAWM, Ibrahim YS, Anuar ST, Govindasamy S, Baharuddin NF (2018). Microplastics analysis in Malaysian marine waters: a field study of Kuala Nerus and Kuantan. Mar Pollut Bull.

[CR45] Kroon F, Motti C, Talbot S, Sobral P, Puotinen M (2018). A workflow for improving estimates of microplastic contamination in marine waters: a case study from North-Western Australia. Environ Pollut.

[CR46] Lenz R, Enders K, Stedmon CA, Mackenzie DMA, Nielsen TG (2015). A critical assessment of visual identification of marine microplastic using Raman spectroscopy for analysis improvement. Mar Pollut Bull.

[CR47] Leslie HA, van der Meulen MD, Kleissen FM, Vethaak AD (2011) Microplastic litter in the Dutch marine environment: providing facts and analysis for Dutch policymakers concerned with marine microplastic litter. (Rapport 1203772-000). Delft / Amsterdam: Deltares / IVM-VU

[CR48] Lima ARA, Costa MF, Barletta M (2014). Distribution patterns of microplastics within the plankton of a tropical estuary. Environ Res.

[CR49] Löder MG, Gerdts G (2015) Methodology used for the detection and identification of microplastics—a critical appraisal. Marine anthropogenic litter. Springer, pp. 201–227. 10.1007/978-3-319-16510-3_8

[CR50] Lusher AL, Burke A, O'Connor I, Officer R (2014). Microplastic pollution in the Northeast Atlantic Ocean: validated and opportunistic sampling. Mar Pollut Bull.

[CR51] Lusher AL, Tirelli V, O’Connor I, Officer R (2015). Microplastics in Arctic polar waters: the first reported values of particles in surface and sub-surface samples. Sci Rep-UK.

[CR52] Maes T, Van der Meulen MD, Devriese LI, Leslie HA, Huvet A, Frère L, Robbens J, Vethaak AD (2017). Microplastic baseline surveys at the water surface and sediments of the North-East Atlantic. Front Mar Sci.

[CR53] Masura J, Baker J, Foster G, Arthur C (2015) Laboratory methods for the analysis of microplastics in the marine environment: recommendations for quantifying synthetic particles in waters and sediments. NOAA Technical Memorandum NOS-OR&R-48, pp. 39

[CR54] MERI (Marine & Environmental Research Institute) (2017) Guide to microplastic identification. Marine & Environmental Research Institute, p 14

[CR55] Miller ME, Kroon FJ, Motti CA (2017). Recovering microplastics from marine samples: a review of current practices. Mar Pollut Bull.

[CR56] Morgana S, Ghigliotti L, Estévez-Calvar N, Stifanese R, Wieckzorek A, Doyle T, Christiansen JS, Faimali M, Garaventa F (2018). Microplastics in the Arctic: a case study with sub-surface water and fish samples off Northeast Greenland. Environ Pollut.

[CR57] Ng KL, Obbard JP (2006). Prevalence of microplastics in Singapore’s coastal marine environment. Mar Pollut Bull.

[CR58] Norén F (2007) Small plastic particles in coastal Swedish waters. KIMO Report, pp. 11

[CR59] Palatinus A, Viršek MK, Kaberi E (2015) DeFishGear protocols for sea surface and beach sediment sampling and sample analysis. pp. 27. http://mio-ecsde.org/wp-content/uploads/2014/12/Protocols-sea-surfacebeach-sediments-Feb15.pdf. Accessed January 2019

[CR60] Pan Z, Guo H, Chen H, Wang S, Sun X, Zou Q, Zhang Y, Lin H, Cai S, Huang J (2019). Microplastics in the northwestern Pacific: abundance, distribution, and characteristics. Sci Total Environ.

[CR61] PlasticsEurope (2018) Annual report 2018 “Plastics – the Facts 2018. An analysis of European latest plastics production, demand and waste data”. https://www.plasticseurope.org/it/resources/market-data. Accessed March 2019

[CR62] Qu X, Su L, Li H, Liang M, Shi H (2018). Assessing the relationship between the abundance and properties of microplastics in water and in mussels. Sci Total Environ.

[CR63] Reisser J, Shaw J, Wilcox C, Hardesty BD, Proietti M, Thums M, Pattiaratchi C (2013). Marine plastic pollution in waters around Australia: characteristics, concentrations, and pathways. PloS One.

[CR64] Rist S, Carney Almroth B, Hartmann NB, Karlsson TM (2018). A critical perspective on early communications concerning human health aspects of microplastics. Sci Total Environ.

[CR65] Sagawa N, Kawaai K, Hinata H (2018). Abundance and size of microplastics in a coastal sea: comparison among bottom sediment, beach sediment, and surface water. Mar Pollut Bull.

[CR66] Saliu F, Montano S, Garavaglia MG, Lasagni M, Seveso D, Galli P (2018). Microplastic and charred microplastic in the Faafu Atoll, Maldives. Mar Pollut Bull.

[CR67] Schnurr REJ, Alboiu V, Chaudhary M, Corbett RA, Quanz ME, Sankar K, Srain HS, Thavarajah V, Xanthos D, Walker TR (2018). Reducing marine pollution from single-use plastics (SUPs): a review. Mar Pollut Bull.

[CR68] Sedlak M, Sutton R, Box C, Sun J, Lin D (2017) Final sampling and analysis plan for microplastic monitoring in San Francisco Bay and adjacent nation marine sanctuaries. SFEI contribution 819. Richmond CA, pp. 136

[CR69] Setälä O, Magnusson K, Lehtiniemi M, Norén F (2016). Distribution and abundance of surface water microlitter in the Baltic Sea: a comparison of two sampling methods. Mar Pollut Bull.

[CR70] Sigma Aldrich (2019) Thermal transitions of homopolymers: glass transition & melting point https://www.sigmaaldrich.com/technical-documents/articles/materials-science/polymer-science/thermal-transitions-of-homopolymers.html (accessed June 2019)

[CR71] Song YK, Hong SH, Jang M, Han GM, Rani M, Lee J, Shim WJ (2015). A comparison of microscopic and spectroscopic identification methods for analysis of microplastics in environmental samples. Mar Pollut Bull.

[CR72] Suaria G, Avio CG, Mineo A, Lattin GL, Magaldi MG, Belmonte G (2016). The Mediterranean plastic soup: synthetic polymers in Mediterranean surface waters. Sci Rep-UK.

[CR73] Sun X, Liang J, Zhu M, Zhao Y, Zhang B (2018). Microplastics in seawater and zooplankton from the Yellow Sea. Environ Pollut.

[CR74] Sutton R, Mason SA, Stanek SK, Willis-Norton E, Wren IF, Box C (2016). Microplastic contamination in the San Francisco Bay, California, USA. Mar Pollut Bull.

[CR75] Syakti AD, Hidayati NV, Jaya YV, Siregar SH, Yude R, Suhendy Asia L, Wong-Wah-Chung P, Doumenq P (2018). Simultaneous grading of microplastic size sampling in the Small Island of Bintan water, Indonesia. Mar Pollut Bull.

[CR76] Tamminga M, Hengstmann E, Fischer EK (2018). Microplastic analysis in the south Funen archipelago, Baltic Sea, implementing manta trawling and bulk sampling. Mar Pollut Bull.

[CR77] Tang G, Liu M, Zhou Q, He H, Chen K, Zhang H, Hu J, Huang Q, Luo Y, Ke H, Chen B, Xu X, Cai M (2018). Microplastics and polycyclic aromatic hydrocarbons (PAHs) in Xiamen coastal areas: implications for anthropogenic impacts. Sci Total Environ.

[CR78] Thompson RC, Olsen Y, Mitchell RP, Davis A, Rowland SJ, John AWG, McGonigle D, Russell AE (2004). Lost at sea: where is all the plastic?. Science.

[CR79] Tunçer S, Artüz OB, Demirkol M, Artüz ML (2018). First report of occurrence, distribution, and composition of microplastics in surface waters of the Sea of Marmara, Turkey. Mar Pollut Bull.

[CR80] van der Hal N, Ariel A, Angel DL (2017). Exceptionally high abundances of microplastics in the oligotrophic Israeli Mediterranean coastal waters. Mar Pollut Bull.

[CR81] Viršek MK, Palatinus A, Koren S, Peterlin M, Horvat P, Kržan A (2016). Protocol for microplastics sampling on the sea surface and sample analysis. JOVEJ Vis Exp.

[CR82] Wang J, Tan Z, Peng J, Qiu Q, Li M (2016). The behaviors of microplastic in the marine environment. Mar Environ Res.

[CR83] Xanthos D, Walker TR (2017). International policies to reduce plastic marine pollution from single-use plastics (plastic bags and microbeads): a review. Mar Pollut Bull.

[CR84] Zhao S, Zhu L, Li D (2015). Microplastic in three urban estuaries, China. Environ Pollut.

[CR85] Zhu J, Zhang Q, Li Y, Tan S, Kang Z, Yu X, Lan W, Cai L, Wang J, Shi H (2019). Microplastic pollution in the Maowei Sea, a typical mariculture bay of China. Sci Total Environ.

[CR86] Zobkov MB, Esiukova EE, Zyubin AY, Samusev IG (2019). Microplastic content variation in water column: the observations employing a novel sampling tool in stratified Baltic Sea. Mar Pollut Bull.

