# Supplementary material for: Microplastics in seawater: sampling strategies, laboratory methodologies, and identification techniques applied to port environment
Source: Environ Sci Pollut Res Int. 2020 Feb 6;27(9):8938–52. doi: 10.1007/s11356-020-07783-8 (PMC7165152; doi:10.1007/s11356-020-07783-8)
Supplement: Supplementary file 1 — (PDF 145 kb) [file 11356_2020_7783_MOESM1_ESM.pdf]

Microplastics in seawater: sampling strategies, laboratory methodologies, and identification techniques applied to port environment

Environmental Science and Pollution Research

Laura Cutroneo[1,*], Anna Reboa[1], Giovanni Besio[2], Franco Borgogno[3], Laura Canesi[1], Susanna Canuto[3], Manuela Dara[1], Francesco Enrile[2], Iskender Forioso[3], Giuseppe Greco[1], Véronique Lenoble[4], Arianna Malatesta[1], Stéphane Mounier[4], Mario Petrillo[1], Ruben Rovetta[2], Alessandro Stocchino[2], Javier Tesan[4], Greta Vagge[1], Marco Capello[1]

* Corresponding author:

DISTAV, University of Genoa, 26 Corso Europa, I-16132, Genoa, Italy

Tel. +39(0)1035358164

Fax +39(0)10352169

E-mail: laura.cutroneo@dipteris.unige.it

Online Resource 1 Complete list of the 74 reviewed research articles

[1] Abayomi OA, Range P, Al-Ghouti MA, Obbard JP, Almeer SH, Ben-Hamadou R (2017) Microplastics in coastal environments of the Arabian Gulf. Mar Pollut Bull 124:181–188. http://dx.doi.org/10.1016/j.marpolbul.2017.07.011

[2] Aytan U, Valente A, Senturk Y, Usta R, Sahin FBE, Mazlum RE, Agirbas E (2016) First evaluation of neustonic microplastics in Black Sea waters. Mar Environ Res 119:22–30. http://dx.doi.org/10.1016/j.marenvres.2016.05.009

[3] Bagaev A, Khatmullina L, Chubarenko I (2018) Anthropogenic microlitter in the Baltic Sea water column. Mar Pollut Bull 129:918–923. http://dx.doi.org/10.1016/j.marpolbul.2017.10.049

[4] Baini M, Fossi MC, Galli M, Caliani I, Campani T, Finoia G, Panti C (2018) Abundance and characterization of microplastics in the coastal waters of Tuscany (Italy): the application of the MSFD monitoring protocol in the Mediterraneand Sea. Mar Pollut Bull 133:543–552. https://doi.org/10.1016/j.marpolbul.2018.06.016

[5] Barrows APW, Neumann CA, Berger ML, Shaw SD (2017) Grab *vs.* neuston tow net: a microplastic sampling performance comparison and possible advances in the field. Anal Methods-UK 9:1446. doi:10.1039/c6ay02387h

[6] Beer S, Garm A, Huwer B, Dierking J, Nielsen TG (2018) No increase in marine microplastic concentration over the last three decades – a case study from the Baltic Sea. Sci Total Environ 621:1272–1279. https://doi.org/10.1016/j.scitotenv.2017.10.101

[7] Bimali Koongolla J, Andrady AL, Terney Pradeep Kumara PB, Gangabadage CS (2018) Evidence of microplastics pollution in coastal beaches and waters in southern Sri Lanka. Mar Pollut Bull 137:277–284. https://doi.org/10.1016/j.marpolbul.2018.10.031

[8] Brandon J, Goldstein M, Ohman MD (2016) Long-term aging and degradation of microplastic particles: comparing in situ oceanic and experimental weathering patterns. Mar Pollut Bull 110:299–308. http://dx.doi.org/10.1016/j.marpolbul.2016.06.048

[9] Cai M, He H, Liu M, Li S, Tang G, Wang W, Huang P, Wei G, Lin Y, Chen B, Hu J, Cen Z (2018) Lost but can’t be neglected: huge quantities of small microplastics hide in the South China Sea. Sci Total Environ 633:1206–1216. https://doi.org/10.1016/j.scitotenv.2018.03.197

[10] Castillo AB, Al-Maslamani I, Obbard JP (2016) Prevalence of microplastics in the marine waters of Qatar. Mar Pollut Bull 111:260–267. http://dx.doi.org/10.1016/j.marpolbul.2016.06.108

[11] Castro RO, Silva ML, Marques MRC, de Araújo FV (2016) Evaluation of microplastics in Jurujuba Cove, Niterói, RJ, Brazil, an area of mussels farming. Mar Pollut Bull 110:555–558. http://dx.doi.org/10.1016/j.marpolbul.2016.05.037

[12] Cincinelli A, Scopetani C, Chelazzi D, Lombardini E, Martellini T, Katsoyiannis A, Fossi MC, Corsolini S (2017) Microplastic in the surface waters of Ross Sea (Antarctica): occurrence, distribution and characterization by FTIR. Chemosphere 175:391–400. http://dx.doi.org/10.1016/j.chemosphere.2017.02.024

[13] Cole M, Webb H, Lindeque PK, Fileman ES, Halsband C, Galloway TS (2014) Isolation of microplastics in biota-rich seawater samples and marine organisms. Sci Rep-UK 4:4528. doi:10.1038/srep04528

[14] Collignon A, Hecq J, Galgani F, Collard F, Goffart A (2014) Annual variation in neustonic micro- and meso-plastic particles and zooplankton in the Bay of Calvi (Mediterranean-Corsica). Mar Pollut Bull 79:293–298. http://dx.doi.org/10.1016/j.marpolbul.2013.11.023

[15] Collignon A, Hecq JH, Galgani F, Voisin P, Collard F, Goffart A (2012) Neustonic microplastic and zooplankton in the North Western Mediterranean Sea. Mar Pollut Bull 64(4):861–864. doi:10.1016/j.marpolbul.2012.01.011

[16] Dai Z, Zhang H, Zhou Q, Tian Y, Chen T, Tu C, Fu C, Luo Y (2018) Occurrence of microplastics in the water column and sediment in an inland sea affected by intensive anthropogenic activities. Environ Pollut 242:1557–1565. https://doi.org/10.1016/j.envpol.2018.07.131

[17] de Lucia GA, Caliani I, Marra S, Camedda A, Coppa S, Alcaro L, Campani T, Giannetti M, Coppola D, Cicero AM, Panti C, Baini M, Guerranti C, Marsili L, Massaro G, Fossi MC, Matiddi M (2014) Amount and distribution of neustonic micro-plastic off the western Sardinian coast (Central-Western Mediterranean Sea). Mar Environ Res 100:10–16. http://dx.doi.org/10.1016/j.marenvres.2014.03.017

[18] Desforges JPW, Galbraith M, Dangerfield N, Ross PS (2014) Widespread distribution of microplastics in subsurface seawater in the NE Pacific Ocean. Mar Pollut Bull 79:94–99. http://dx.doi.org/10.1016/j.marpolbul.2013.12.035

[19] Dubaish F, Liebezeit G (2013) Suspended microplastics and black carbon particles in the Jade system, Southern North Sea. Water Air Soil Poll 224(2):1–8. doi:10.1007/s11270-012-1352-9

[20] Edson E, Patterson M (2015 MantaRay: a novel autonomous microplastic sensor for determining particle concentrations in marine ecosystems. Research, Innovation and Scholarship Expo. Category: Engineering and Technology. Abstract ID# 580. Boston (MA; USA)

[21] Enders K, Lenz R, Stedmon CA, Nielsen TG (2015). Abundance, size and polymer composition of marine microplastics ≥10 μm in the Atlantic Ocean and their modelled vertical distribution. Mar Pollut Bull 100:70–81. http://dx.doi.org/10.1016/j.marpolbul.2015.09.027

[22] Eriksen M, Liboiron M, Kiessling T, Charron L, Alling A, Lebreton L, Richards H, Roth B, Ory NC, Hidalgo-Ruz V, Meerhoff E, Box C, Cummins A, Thiel M (2018) Microplastic sampling with the AVANI trawl compared to two neuston trawls in the Bay of Bengal and South Pacific. Mar Pollut Bull 232:430–439. https://doi.org/10.1016/j.envpol.2017.09.058

[23] Erni-Cassola G, Gibson MI, Thompson RC, Christie-Oleza JA (2017) Lost, but found with Nile Red: a novel method for detecting and quantifying small microplastics (1 mm to 20 µm) in environmental samples. Environ Sci Technol 51:13641–13648. doi:10.1021/acs.est.7b04512

[24] Faur F, Saini C, Potter G, Galgani F, De Alencastro LF, Hagmann P (2015) An evaluation of surface micro- and mesoplastic pollution in pelagic ecosystems of the Western Mediterranean Sea. Environ Sci Pollut Res 22(16):12190–12197. http://dx.doi.org/10.1007/s11356-015-4453-3

[25] Figueiredo GM, Vianna TMP (2018) Suspended microplastics in a highly polluted bay: abundance, size, and availability for mesozooplankton. Mar Pollut Bull 135:256–265. https://doi.org/10.1016/j.marpolbul.2018.07.020

[26] Frère L, Paul-Pont I, Moreau J, Soudant P, Lambert C, Huvet A, Rinnert E (2016) A semi-automated Raman micro-spectroscopy method for morphological and chemical characterizations of microplastic litter. Mar Pollut Bull 113:461–468. http://dx.doi.org/10.1016/j.marpolbul.2016.10.051

[27] Frias JPGL, Otero V, Sobral P (2014) Evidence of microplastics in samples of zooplankton from Portuguese coastal waters. Mar Environ Res 95:89–95. http://dx.doi.org/10.1016/j.marenvres.2014.01.001

[28] Gajšt T, Bizjak T, Palatinus A, Liubartseva S, Kržan A (2016) Sea surface microplastics in Slovenian part of the northern Adriatic. Mar Pollut Bull 113:392–399. http://dx.doi.org/10.1016/j.marpolbul.2016.10.031

[29] Gallagher A, Rees A, Rowe R, Stevens J, Wright P (2016) Microplastics in the Solent estuarine complex, UK: an initial assessment. Mar Pollut Bull 102:243–249. http://dx.doi.org/10.1016/j.marpolbul.2015.04.002

[30] Gorokhova E (2015) Screening for microplastic particles in plankton samples: how to integrate marine litter assessment into existing monitoring programs? Mar Pollut Bull 99:271–275. http://dx.doi.org/10.1016/j.marpolbul.2015.07.056

[31] Green DS, Kregting L, Boots B, Blockley DJ, Brickle P, da Costa M, Crowley Q (2018) A comparison of sampling methods for seawater microplastics and a first report of the microplastic litter in coastal waters of Ascension and Falkland Islands. Mar Pollut Bull 137:695–701. https://doi.org/10.1016/j.marpolbul.2018.11.004

[32] Güven O, Gökdağ K, Jovanović B, Kıdeyş AE (2017) Microplastic litter composition of the Turkish territorial waters of the Mediterranean Sea, and its occurrence in the gastrointestinal tract of fish. Environ Pollut 223:286–294. http://dx.doi.org/10.1016/j.envpol.2017.01.025

[33] Hall NM, Berry KLE, Rintoul L, Hoogenboom MO (2015) Microplastic ingestion by scleractinian corals. Mar Biol 162(3):725–732. doi:10.1007/s00227-015-2619-7

[34] Isobe A (2016) Percentage of microbeads in pelagic microplastics within Japanese coastal waters. Mar Pollut Bull 110:432–437. http://dx.doi.org/10.1016/j.marpolbul.2016.06.030

[35] Isobe A, Uchida K, Tokai T, Iwasaki S (2015) East Asian seas: a hot spot of pelagic microplastics. Mar Pollut Bull 101:618–623. http://dx.doi.org/10.1016/j.marpolbul.2015.10.042

[36] Kang JH, Kwon OY, Lee KW, Song YK, Shim WJ (2015) Marine neustonic microplastics around the southeastern coast of Korea. Mar Pollut Bull 96:304–312. http://dx.doi.org/10.1016/j.marpolbul.2015.04.054

[37] Kanhai LDK, Gårdfeldt K, Lyashevska O, Hassellöv M, Thompson RC, O’Connor I (2018) Microplastics in sub-surface waters of the Arctic Central Basin. Mar Pollut Bull 130:8–18. https://doi.org/10.1016/j.marpolbul.2018.03.011

[38] Khalik WMAWM, Ibrahim YS, Anuar ST, Govindasamy S, Baharuddin NF (2018) Microplastics analysis in Malaysian marine waters: a field study of Kuala Nerus and Kuantan. Mar Pollut Bull 135:451–457. https://doi.org/10.1016/j.marpolbul.2018.07.052

[39] Kroon F, Motti C, Talbot S, Sobral P, Puotinen M (2018) A workflow for improving estimates of microplastic contamination in marine waters: a case study from North-Western Australia. Environ Pollut 238:26–38. https://doi.org/10.1016/j.envpol.2018.03.010

[40] Lenz R, Enders K, Stedmon CA, Mackenzie DMA, Nielsen TG (2015) A critical assessment of visual identification of marine microplastic using Raman spectroscopy for analysis improvement. Mar Pollut Bull 100:82–91. http://dx.doi.org/10.1016/j.marpolbul.2015.09.026

[41] Lima ARA, Costa MF, Barletta M (2014) Distribution patterns of microplastics within the plankton of a tropical estuary. Environ Res 132:146–155. http://dx.doi.org/10.1016/j.envres.2014.03.031

[42] Lusher AL, Burke A, O'Connor I, Officer R (2014) Microplastic pollution in the Northeast Atlantic Ocean: validated and opportunistic sampling. Mar Pollut Bull 88(1):325–333. http://dx.doi.org/10.1016/j.marpolbul.2014.08.023

[43] Lusher AL, Tirelli V, O'Connor I, Officer R (2015) Microplastics in Arctic polar waters: the first reported values of particles in surface and sub-surface samples. Sci Rep-UK 5:14947. doi:10.1038/srep14947

[44] Maes T, Van der Meulen MD, Devriese LI, Leslie HA, Huvet A, Frère L, Robbens J, Vethaak AD (2017) Microplastic baseline surveys at the water surface and sediments of the North-East Atlantic. Front Mar Sci 4:135. doi:10.3389/fmars.2017.00135

[45] Morgana S, Ghigliotti L, Estévez-Calvar N, Stifanese R, Wieckzorek A, Doyle T, Christiansen JS, Faimali M, Garaventa F (2018) Microplastics in the Arctic: a case study with sub-surface water and fish samples off Northeast Greenland. Environ Pollut 242:1078–1086. https://doi.org/10.1016/j.envpol.2018.08.001

[46] Ng KL, Obbard JP (2006) Prevalence of microplastics in Singapore's coastal marine environment. Mar Pollut Bull 52:761−767. doi:10.1016/j.marpolbul.2005.11.017

[47] Norén F (2007) Small plastic particles in Coastal Swedish waters. KIMO Report, pp. 11.

[48] Palatinus A, Viršek MK, Kaberi E (2015) DeFishGear protocols for sea surface and beach sediment sampling and sample analysis. pp. 27. http://mio-ecsde.org/wp-content/uploads/2014/12/Protocols-sea-surfacebeach-sediments-Feb15.pdf

[49] Pan Z, Guo H, Chen H, Wang S, Sun X, Zou Q, Zhang Y, Lin H, Cai S, Huang J (2019) Microplastics in the Northwestern Pacific: abundance, distribution, and characteristics. Sci Total Environ 650:1913−1922. https://doi.org/10.1016/j.scitotenv.2018.09.244

[50] Qu X, Su L, Li H, Liang M, Shi H (2018) Assessing the relationship between the abundance and properties of microplastics in water and in mussels. Sci Total Environ 621:679−686. https://doi.org/10.1016/j.scitotenv.2017.11.284

[51] Reisser J, Shaw J, Wilcox C, Hardesty BD, Proietti M, Thums M, Pattiaratchi C (2013) Marine plastic pollution in waters around Australia: characteristics, concentrations, and pathways. PloS One 8(11). doi: e80466

[52] Sagawa N, Kawaai K, Hinata H (2018) Abundance and size of microplastics in a coastal sea: comparison among bottom sediment, beach sediment, and surface water. Mar Pollut Bull 133:532−542. https://doi.org/10.1016/j.marpolbul.2018.05.036

[53] Saliu F, Montano S, Garavaglia MG, Lasagni M, Seveso D, Galli P (2018) Microplastic and charred microplastic in the Faafu Atoll, Maldives. Mar Pollut Bull 136:464−471. https://doi.org/10.1016/j.marpolbul.2018.09.023

[54] Setälä O, Magnusson K, Lehtiniemi M, Norén F (2016) Distribution and abundance of surface water microlitter in the Baltic Sea: a comparison of two sampling methods. Mar Pollut Bull 110(1):177−183. http://dx.doi.org/10.1016/j.marpolbul.2016.06.065

[55] Shim WJ, Song YK, Hong SH, Jang M (2016) Identification and quantification of microplastics using Nile Red staining. Mar Pollut Bull 113:469−476. http://dx.doi.org/10.1016/j.marpolbul.2016.10.049

[56] Song YK, Hong SH, Jang M, Kang JH, Kwon OY, Han GM, Shim WJ (2014) Large accumulation of micro-sized synthetic polymer particles in the sea surface microlayer. Environ Sci Technol 48:9014–9021. http://dx.doi.org/10.1021/es501757s

[57] Song YK, Hong SH, Jang M, Han GM, Rani M, Lee J, Shim WJ (2015) A comparison of microscopic and spectroscopic identification methods for analysis of microplastics in environmental samples. Mar Pollut Bull 93:202–209. doi:10.1016/j.marpolbul.2015.01.015

[58] Stöhr R (2016) Microplastic in the Sea. A research approach by One Earth – One Ocean. pp. 13. https://oneearth-oneocean.com/wp-content/uploads/Microplastic-in-the-sea-report-I.pdf

[59] Suaria G, Avio CG, Mineo A, Lattin GL, Magaldi MG, Belmonte G (2016) The Mediterranean plastic soup: synthetic polymers in Mediterranean surface waters. Sci Rep-UK 6:37551. doi:10.1038/srep37551

[60] Sun X, Liang J, Zhu M, Zhao Y, Zhang B (2018) Microplastics in seawater and zooplankton from the Yellow Sea. Environ Pollut 242:585−595. doi:10.1016/j.envpol.2018.07.014

[61] Sutton R, Mason SA, Stanek SK, Willis-Norton E, Wren IF, Box C (2016) Microplastic contamination in the San Francisco Bay, California, USA. Mar Pollut Bull 109:230–235. http://dx.doi.org/10.1016/j.marpolbul.2016.05.077

[62] Syakti AD, Bouhroum R, Hidayati NV, Koenawan CJ, Boulkamh A, Sulistyo I, Lebarillier S, Akhlus S, Doumenq P, Wong-Wah-Chung P (2017) Beach macro-litter monitoring and floating microplastic in a coastal area of Indonesia. Mar Pollut Bull 122:217−225. http://dx.doi.org/10.1016/j.marpolbul.2017.06.046

[63] Syakti AD, Hidayati NV, Jaya YV, Siregar SH, Yude R, Suhendy Asia L, Wong-Wah-Chung P, Doumenq P (2018) Simultaneous grading of microplastic size sampling in the Small Island of Bintan water, Indonesia. Mar Pollut Bull 137:593−600. https://doi.org/10.1016/j.marpolbul.2018.11.005

[64] Tamminga M, Hengstmann E, Fischer EK (2018) Microplastic analysis in the South Funen Archipelago, Baltic Sea, implementing manta trawling and bulk sampling. Mar Pollut Bull 128:601−608. http://doi.org/10.1016/j.marpolbul.2018.01.066

[65] Tang G, Liu M, Zhou Q, He H, Chen K, Zhang H, Hu J, Huang Q, Luo Y, Ke H, Chen B, Xu X, Cai M (2018) Microplastics and polycyclic aromatic hydrocarbons (PAHs) in Xiamen coastal areas: implications for anthropogenic impacts. Sci Total Environ 634:811−820. https://doi.org/10.1016/j.scitotenv.2018.03.336

[66] Thompson RC, Olsen Y, Mitchell RP, Davis A, Rowland SJ, John AWG, McGonigle D, Russell AE (2004) Lost at sea: where is all the plastic? Science 304:838 p. doi:10.1126/science.1094559

[67] Tsang YY, Mak CW, Liebich C, Lam SW, Sze ETP, Chan KM (2017) Microplastic pollution in the marine waters and sediments of Hong Kong. Mar Pollut Bull 115:20−28. http://dx.doi.org/10.1016/j.marpolbul.2016.11.003[

[68] Tunçer S, Artüz OB, Demirkol M, Artüz ML (2018) First report of occurrence, distribution, and composition of microplastics in surface waters of the Sea of Marmara, Turkey. Mar Pollut Bull 135:283−289. https://doi.org/10.1016/j.marpolbul.2018.06.054

[69] van der Hal N, Ariel A, Angel DL (2017) Exceptionally high abundances of microplastics in the oligotrophic Israeli Mediterranean coastal waters. Mar Pollut Bull 116:151–155. http://dx.doi.org/10.1016/j.marpolbul.2016.12.052

[70] Viršek MK, Palatinus A, Koren S, Peterlin M, Horvat P, Kržan A (2016) Protocol for microplastics sampling on the sea surface and sample analysis. JOVEJ Vis Exp 118:55161. doi:10.3791/55161

[71] Zhao S, Zhu L, Li D (2015) Microplastic in three urban estuaries, China. Environ Pollut 206:597–604. http://dx.doi.org/10.1016/j.envpol.2015.08.027

[72] Zhao S, Zhu L, Wang T, Li D (2014) Suspended microplastics in the surface water of the Yangtze Estuary System, China: first observations on occurrence, distribution. Mar Pollut Bull 86:562–568. http://dx.doi.org/10.1016/j.marpolbul.2014.06.032

[73] Zhu J, Zhang Q, Li Y, Tan S, Kang Z, Yu X, Lan W, Cai L, Wang J, Shi H (2019) Microplastic pollution in the Maowei Sea, a typical mariculture bay of China. Sci Total Environ 658:62–68. https://doi.org/10.1016/j.scitotenv.2018.12.192

[74] Zobkov MB, Esiukova EE, Zyubin AY, Samusev IG (2019) Microplastic content variation in water column: the observations employing a novel sampling tool in stratified Baltic Sea. Mar Pollut Bull 138:193–205
